# Knowledge of Nipah Virus and Associated Factors Among Rural Population in Bangladesh: Evidence From a Community‐Based Cross‐Sectional Study

**DOI:** 10.1002/hsr2.73429

**Published:** 2026-10-27

**Authors:** Tamsel Ahammed, Md. Ahsan Habib, Md. Moshiuzzaman Adnan, Sabrina Zaman, Natasha Kundu, Mst Sumaya Binta Bilkis, Kazi Nazifa Anzum Setu, Md. Lokman Hossain, Md. Rakibul Hassan Tarek, Susmita Roy Chowdhury, Md. Shamimuzzaman, Rajib Kanti Roy

**Affiliations:** ^1^ Department of Nutrition and Food Technology, Faculty of Applied Science and Technology Jashore University of Science and Technology Jashore Bangladesh; ^2^ Department of Nutrition and Food Engineering, Faculty of Health and Life Sciences Daffodil International University Birulia Dhaka Bangladesh; ^3^ Department of Microbiology, Faculty of Biological Science and Technology Jashore University of Science and Technology Jashore Bangladesh; ^4^ National Control Laboratory, Directorate General of Drug Administration Ministry of Health and Family Welfare Dhaka Bangladesh

**Keywords:** date palm sap, knowledge, Nipah virus, rural population, zoonotic disease

## Abstract

**Background and Aims:**

Nipah virus (NiV) is a highly pathogenic zoonotic threat and posing serious public health risks in Bangladesh due to its high fatality rate, high transmissibility, and the lack of particular licensed vaccinations.

**Methods:**

A community‐based cross‐sectional study was carried out among 415 adult rural respondents. Data were collected using a paper‐based structured questionnaire consisting of respondents' sociodemographic characteristics and knowledge‐related questions regarding NiV. Participants' sociodemographic characteristics and knowledge level were summarized using descriptive statistics, chi‐square tests, and multivariable logistic regression. The statistical significance level was set at *p* < 0.05, with 95% confidence intervals (CIs).

**Results:**

The mean age of participants was 37.56 ± 14.63 years, and 66.5% were male. Although 71.1% of participants had heard of the NiV, only 16.4% demonstrated good knowledge (≥ 65% of total score). The mean knowledge score was 7.99 ± 3.86. Knowledge was particularly limited regarding routes of transmission, including person‐to‐person transmission, clinical manifestations, treatment availability, and the ineffectiveness of antibiotics. In the adjusted analysis, participants aged 25–34 years (AOR = 2.85, 95% CI: 1.13–7.63), females (AOR = 2.73, 95% CI: 1.09–7.09), and those with graduation or higher education (AOR = 10.70, 95% CI: 2.10–67.70) were significantly more likely to have good knowledge. Conversely, participants who consumed fresh date palm sap had significantly lower odds of good knowledge (AOR = 0.34, 95% CI: 0.17–0.68).

**Conclusion:**

The rural respondents in this study area demonstrated inadequate awareness of the NiV. Participants' understanding of disease symptoms, treatment limitations, disease transmission, and prophylactic measures was remarkably poor. To prevent NiV transmission and outbreaks in Bangladesh, these findings highlight the significance of targeted, culturally appropriate, and community‐based health education programs that focus on safe date palm sap consumption, early symptom recognition, and other effective preventive measures.

## 1 Introduction

Human Nipah virus (NiV) infection is a zoonotic disease that has raised major public health challenges in South and Southeast Asia [1]. This highly contagious virus was initially discovered among pig breeders in Malaysia and Singapore between 1998 and 1999 [2, 3]. Since then, NiV infection has grown to be a major global health concern. NiV, one of the most lethal emerging zoonotic viruses, is a member of the genus *Henipavirus* in the family *Paramyxoviridae* [4]. Considering the nature of NiV, its high mortality rate, risk of being transmitted from animals to humans, and the potential danger of a worldwide epidemic, World Health Organization (WHO) declared NiV as an important virus in 2018 [5, 6]. The inability to foresee NiV outbreaks, along with a lack of antiviral treatments and vaccinations, poses a significant health risk to humanity. Infection in humans is caused by direct or indirect contact with infected bats or animals, ingestion of contaminated food, or close contact with an infected individual. Fruit bats of the genus *Pteropus* serve as the natural reservoir of NiV [7]. Clinical manifestations of this infection widely vary from severe encephalitis and respiratory distress to high fever; however, infections sometimes can remain completely asymptomatic. Case mortality rates have been estimated to be between 40% and 75%, with some outbreaks reaching as high as 90% [4, 5]. Since there is no approved human vaccine or targeted antiviral treatment for this infection, effective risk communication and early diagnosis of disease are the main approaches to disease control [4, 8]. NiV was initially linked to an encephalitis epidemic in Bangladesh from 2001 [9], and remains one of the most country's recurring outbreaks. Since then, 20 districts in Bangladesh have seen numerous NiV epidemic outbreaks, all of which took place between December and May during the winter season, when date palm sap is collected and eaten [10]. By 2021, a country‐wide national surveillance in Bangladesh had identified 322 verified human cases with an average case mortality rate near 71%, and the most recent studies indicate a continuous rise in the cumulative case count [11]. To date, Bangladesh has recorded a total of 348 cases of NiV infection, including 250 deaths, resulting in an overall case fatality rate of 72%. Among these cases, 29% were affected by person‐to‐person infection, while nearly half of them (*n* = 162) were the primary cases with a confirmed history of consuming either raw or fermented date palm sap. In 2025, there were four fatal cases found in Bangladesh that were confirmed through laboratory tests. On February 3, 2026, the International Health Regulations National Focal Point (IHR NFP) for Bangladesh reported one case of Nipah infection to WHO. Since 2016, Bangladesh has been experiencing less than 10 cases of NiV infection every year with the exception of 2023 that witnessed 14 cases. However, the spread of NiV through human‐to‐human contact is rare now [12]. When compared with other countries, Bangladesh experiences a significantly higher mortality rate (72%) due to NiV infection than that of Malaysia (53%), the Philippines (29%), and Singapore (21%) [13]. In Bangladesh, virus transmission primarily occurs through two major routes: when one consumes raw date palm sap contaminated with bat saliva, and comes in close contact by family caregivers providing direct care to infected patients both at home and in hospital settings [4, 11].

Climate and human factors had a significant impact on NiV epidemiology. Deforestation and farming operations have destroyed habitat, forcing Pteropus bats to reside close to human populations. The bats might be feeding in agricultural and rural settings, which raises the likelihood of a zoonotic spread. In Bangladesh, deforestation has altered the bats' natural environment, causing them to come into more contact with humans in rural regions where raw date palm sap is extracted [14]. Molecular research has yielded information about the virulence and evolution of NiV. The two main forms of NiV genetic variants have been identified: one from Bangladesh and India, which has recently produced outbreaks, and the second from Malaysia, which caused the initial outbreak [15, 16]. The Bangladeshi strain has shown enhanced virulence and genetic heterogeneity, with death rates in some outbreaks exceeding 75%. Approaches for NiV surveillance and diagnosis are significantly impacted by these molecular differences [11, 17]. Public health officials can study the evolution of the virus and possible shifts in the dynamics of transmission with the aid of genomic surveillance, which could guide the creation of vaccines and treatments for NiV [17, 18]. Preventing future epidemics requires an awareness regarding the factors that contribute to NiV spread [10]. Attention should be paid to biological and environmental changes, including deforestation and human encroachment into wildlife areas, to minimize the NiV vulnerability [1].

According to the Food and Agriculture Organization (FAO), harvesting date palm sap represents an extensive economic and cultural heritage in rural Bengal. Traditionally, this sap of sweet, earthy taste is widely consumed fresh in the morning or is converted to popular winter sweets such as *Gur* (jaggery) [19]. When harvesting season begins in the winter, harvesters shave those date palm tree trunks and bats (*Pteropus*) are attracted to the sweet oozing sap. If sap collection pots are open at night, bats contaminate the raw sap with their saliva, urine, and feces. Then humans become infected if they consume this raw, unboiled date palm sap [20]. It was observed that Nipah cases were 25 times more likely than controls to have consumed raw date palm sap [21]. Southwestern Bangladesh, including Jashore, Jhenaidah, Magura, and Kushtia, is vastly enriched with wild date palm trees; hence, raw sap consumption is also highly common there. Therefore, epidemiologically, these areas attract researchers intensely due to recurrent bat‐borne NiV outbreaks [8].

Literature search clearly demonstrated that numerous studies [8, 22, 23, 24, 25] have been carried out in Bangladesh to assess the knowledge, attitudes, and practices level among the respondents regarding NiV. These studies revealed serious concern in community understanding regarding the disease's symptoms, modes of transmission, seasonality, geographic spread, and prevention strategies. Furthermore, it has been demonstrated that knowledge of the NiV is significantly predicted by educational attainment, occupation, age, gender, and geographic area of the study [23, 24]. However, no study was conducted to assess the knowledge level regarding NiV infection among the rural population in Bangladesh, specifically in the Jashore district. Since rural environment, culture, health information sources, educational facilities, and socioeconomic status may distinctly influence overall knowledge and perception, it is imperative to conduct a community‐specific study which can complement national‐level findings by identifying existing local knowledge gaps and particular population groups that may need focused health‐education interventions.

Understanding the variations within such a community‐specific rural setting, which might be overlooked by the previous nationwide studies, can assist public health personnel to prepare a plan for possible future NiV outbreaks and aid in the development of suitable health education programs in Bangladesh. Bangladesh has seen almost yearly outbreaks of NiV since it was first reported there in 2001. Therefore, this study aims to investigate the knowledge level regarding NiV infection and identify related sociodemographic variables among the rural population of Jashore, Bangladesh. The results of this research will contribute to the development of country‐level baseline information for appropriate and culturally adapted health education programs, national prevention campaigns, and monitoring initiative regarding NiV infection.

## 2 Methods and Methodology

### 2.1 Study Duration, Study Design, and Setting

This study was designed as a community‐based cross‐sectional study to evaluate the knowledge regarding NiV and the associated factors with that knowledge among the rural residents of Jashore district, Bangladesh. The data were collected from March 2026 to June 2026 in the rural area of Jashore, the 13th largest district comprising eight upazila (Abhaynagar, Bagherpara, Chaugaccha, Jhikargacha, Keshabpur, Jashore Sadar, Manirampur, and Sharsha) with an area of 2606.94 sq. km situated in the southwestern part of Bangladesh [26]. The Jashore region experiences extremely cool and dry weather during the winter season. Daytime high temperatures typically range from 25°C to 28°C (77°F–83°F), while nightly lows typically range from 12°C to 14°C (53°F–57°F). However, extreme cold wave conditions can bring temperatures as low as 7.8°C. Southwestern Bangladesh, particularly the Jashore region, is famous for a huge number of wild date trees with consequential extensive harvesting and consumption of raw date sap [27]. This is why people living in this area are at high risk of coming in contact with bat‐borne NiV. This study area is a potential risk zone for NiV infection because of its previous history of recurrent outbreaks. In April 2008, the case fatality rate was 50% in Satkhira‐Jashore area [28]. Furthermore, recently, due to the risk of NiV in Jashore district, an active surveillance site has also been implemented in Jashore 250‐bedded Hospital [29]. These factors justified selecting the rural area of Jashore district as an appropriate setting for this study aiming to evaluate respondents' knowledge of the NiV and its preventive measures. The study area of this research is presented in Figure 1.

**Figure 1:**
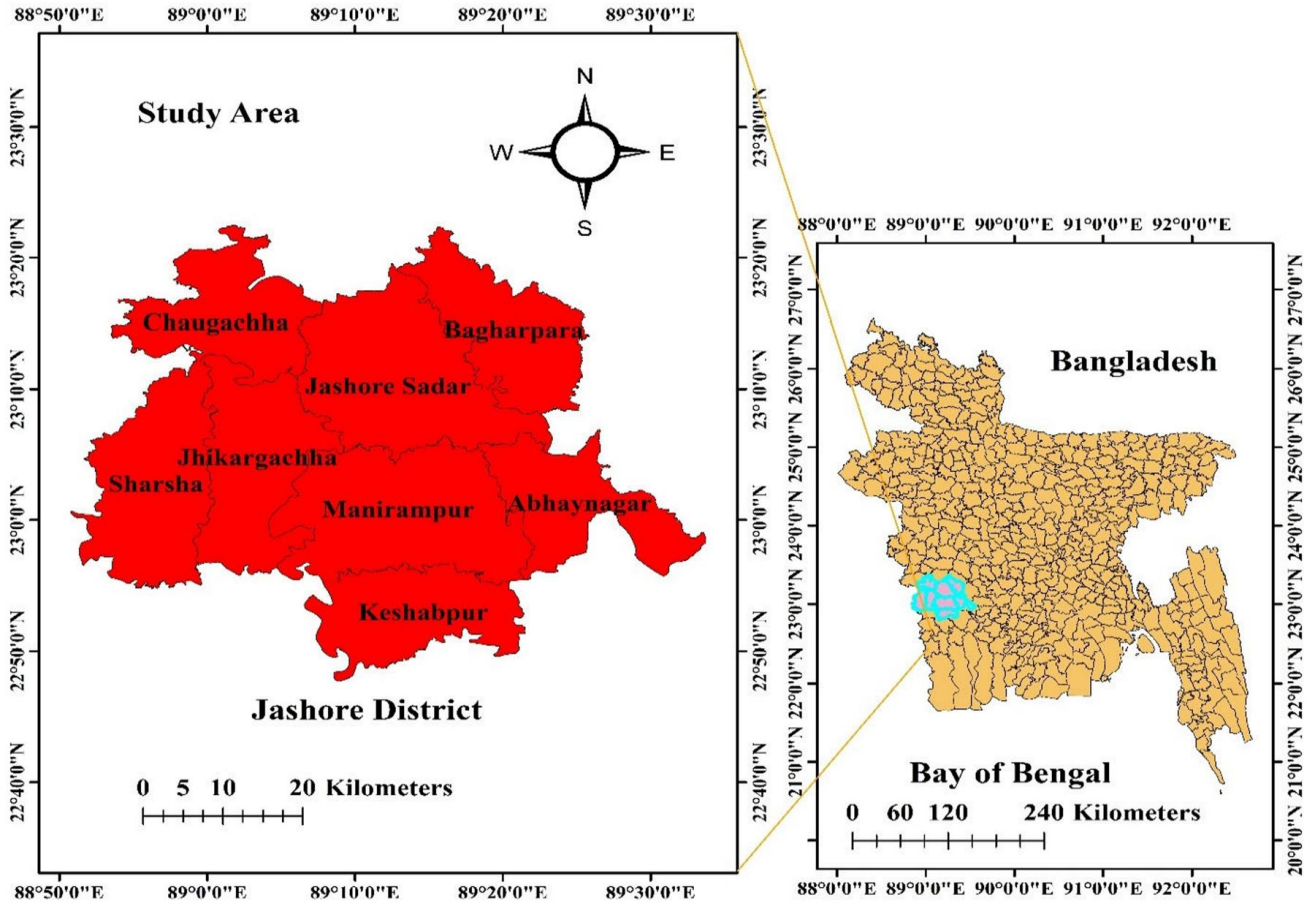
Study area of this research.

### 2.2 Study Population

The study sample was comprised of adult residents living in the selected rural areas in Jashore district. The study population inclusion criteria were: (i) respondents with age of ≥ 18 years, (ii) living in the study area for ≥ 10 years, (iii) being able to communicate in the Bengali language, (iv) being interested to give written consent, (v) living in the rural area of Jashore district. Respondents were excluded under the following criteria: (i) respondents aged < 18 years, (ii) living in the study area for < 10 years, (iii) being severely ill during the survey period, (iv) having cognitive impairment or mental disability, (v) transient visitors in the study area, (vi) respondents with incomplete questionnaires, and (vii) not willing to provide written consent. A detailed methodological overview of this study is presented in Figure 2.

**Figure 2:**
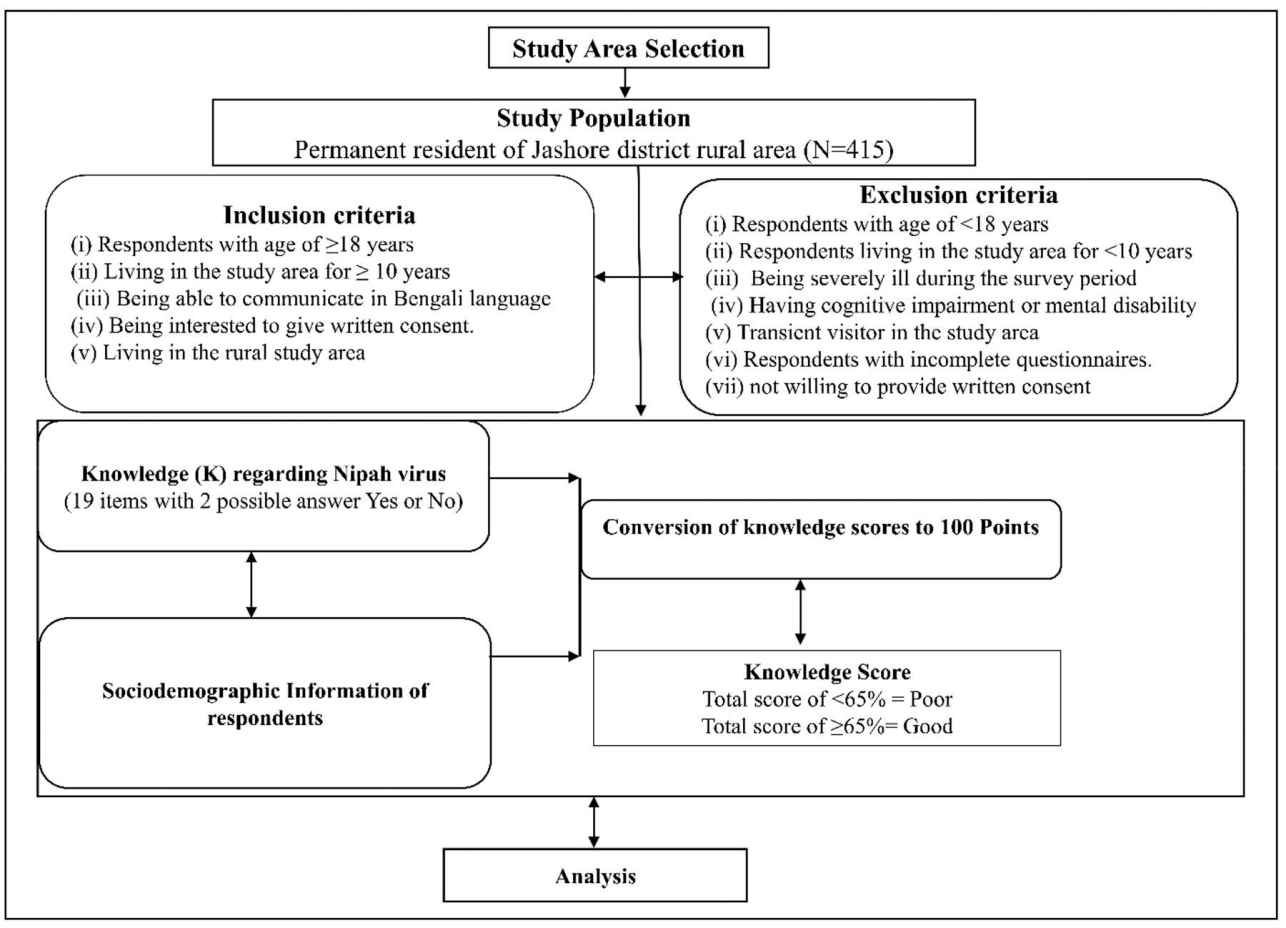
Methodological overview of this study.

### 2.3 Sample Size and Sampling Procedure

A multistage sampling approach was used to recruit participants from selected rural areas of Jashore, thereby facilitating coverage of the target study population within the selected sampling areas. At each sampling stage, eligible participants were identified according to the predefined inclusion and exclusion criteria. In the first stage, Jashore district was selected using purposive sampling, as this study area was previously identified as a potential risk zone for NiV infection, as raw date palm sap is highly consumed during the winter season. In the second stage, eight upazilas, namely Abhaynagar, Bagherpara, Chaugaccha, Jhikargacha, Keshabpur, Jashore Sadar, Manirampur, and Sharsha, were selected from the study area. From each upazila, approximately 60 households were systematically sampled, and one eligible participant per household was randomly selected to ensure adequate respondents' coverage across all regions in Jashore district. Of the 480 respondents initially selected, 475 respondents were eligible and recruited, and 456 respondents completed the interview. After data screening and quality checks, 415 participants were included in the final analysis. The participant selection and inclusion process were reviewed to ensure consistency in the reported numbers. The sampling design and any clustering procedures were described according to the methods actually implemented. The sampling interval was calculated by dividing the total number of eligible households in each selected community by the required number of households to be sampled [30].

The sample size for this study was calculated by using the Cochran's formula [23].

(1) $$n=\frac{z^{2}p\;(1-p)}{d^{2}}.$$

Here, *n* is the required sample size, *z* is the standard normal deviation at the 95% confidence level (1.96), *p* is the expected prevalence of the outcome (0.50), and *d* is the margin of error (0.05). According to this equation, the minimum required sample size was 384. Then, to compensate for potential non‐responses, a total of 456 households were systematically sampled using an expected 95% response rate. However, excluding the incomplete data, only 415 respondents were finally included in the study. Since the study included one respondent from each selected household, cluster effect was not present, dismissing the need for cluster‐based statistical adjustments.

### 2.4 Questionnaire Development

A structured, pre‐tested and self‐administered questionnaire was developed from previously published literature on the epidemiological information and preventive measures of NiV infection, with particular focus on knowledge, attitude and practices (KAP) studies in Bangladesh [23] and South Asia [31]. The questionnaire tool was adjusted to align with the objectives and the epidemiological conditions of rural areas of Bangladesh. The questionnaire was phrased in simple language to minimize interviewer and respondent bias. The draft questionnaire was reviewed by public health, epidemiology, and infectious disease expert for questionnaire validity and contextual relevance. Then, the questionnaire was translated from English to Bengali for easy communication with the respondents. The Bengali and English version of the questionnaire was thoroughly checked by the supervisor. Before conducting the final survey, the questionnaire was pilot‐tested with 30 respondents from the sampling area to check the clarity, acceptability, and effectiveness of the questionnaire. Feedback from the pilot test was carefully addressed to maintain the comprehensiveness of the data collection method. The reliability of the questionnaire was also evaluated with Cronbach's alpha during the pilot test, which was 0.74, indicating an acceptable level [32]. The calculation formula for Cronbach's alpha is suitable for scales both with dichotomous scores (“Yes”/“No” or “Correct”/“Incorrect”) and polytomous scores (“Never”/“Sometimes”/“Always”). If all the dichotomous items are scored as “1” or “0,” then the formula for Kuder–Richardson Formula 20 and Cronbach's alpha is equivalent [33].

### 2.5 Sociodemographic and Health‐Related Information

This section consisted of 10 questions including the age group (< 25 years, 25–34 years, 35–44 years, 45–54 years, > 55 years), gender (male and female), education (no formal education, primary, secondary, graduation and above), marital status (single and married), occupation (laborer, farmer, housewife, businessman, student, govt. job, and unemployed), monthly income (< 10,000 BDT, 10,000–20,000 BDT, and > 20,000 BDT), BMI category (normal weight, underweight, overweight, and obesity), smoking status (never smoke and current smoker), chronic disease (absent and present) and fresh date juice consumption (no and yes).

### 2.6 Knowledge Regarding NiV

This section of the questionnaire comprised 19 close‐ended questions to evaluate the respondents' knowledge about NiV. Every question had two possible answers: “Yes” or “No.” Answers were coded as “1” for “Yes” and “0” for “No.” Reverse‐coding was not applied because the questions employed scoring based on prespecified answers rather than the direction of possible answers. Each question with a score of “1” indicates the scientifically correct answer; on the other hand, a score of “0” indicates that the answer is incorrect. Therefore, the knowledge scores can possibly range from 0 to 19 that means an individual can score as low as 0 to as high as 19 based on his/her ability to correct right number of answers. Higher scores indicate an individual has a better understanding of NiV infection. The knowledge scores were then multiplied by 100. Then, the scores were categorized into “good” and “poor” knowledge using a 65% cut‐off according to Hossain et al. (2025) [23]. Respondents who correctly answered greater than or equal to 13 questions means they scored ≥ 65% of the total possible score—were categorized as having “good” knowledge and whereas, those correctly answered lower than equal to 12 questions means they scored < 65% of the total possible scores—were classified as having “poor” knowledge. The questions included fundamental concepts and awareness regarding NiV such as its severity, modes of transmission (bats as the natural source, contaminated raw date juice), clinical diagnosis, treatment availability, transmission through bat saliva and excreta, and preventive measures such as covering collected juice pots to block bat contamination. Table 1 represents the respondent's knowledge level assessment method for this study.

**Table 1:** Respondent knowledge level assessment method.

Consumer knowledge level assessment		
Category	Knowledge score range (0–19)	Percentage
Poor knowledge	0 to ≤ 12	< 65% of total score
Good knowledge	≥ 13	≥ 65% of total score

### 2.7 Data Collection Procedure

During this study, data were collected through face‐to‐face interviews using the Bengali questionnaire. Before data collection was initiated, research assistants were trained on the study objectives, survey method, ethical conditions, and informed consent procedures, with specific attention on administering questions consistently to limit bias. Respondents were approached at their households and informed about the study objectives and data collection methods. Written informed consent was obtained before each interview, which was conducted in a private setting to protect confidentiality. Completed questionnaires were reviewed for missing information and consistency before data entry. The lead author of this research checked all the data before and after data entry to ensure consistency of collected data.

### 2.8 Statistical Analysis

2.8.1

The study participants' sociodemographic characteristics (age group, gender, education, marital status, occupation, monthly income, smoking status, chronic disease, fresh date juice consumption), and knowledge‐related responses were all described using frequencies, percentages, and interquartile ranges (IQRs). Data were analyzed using SPSS (Statistical Package for the Social Sciences) version 26 (IBM Corp., Armonk, NY, USA). The entered data were then checked for data quality and miscoding before statistical analysis. To achieve the objectives of the study, both descriptive and multivariable logistic regression was employed. The associations between sociodemographic variables and knowledge levels were first checked with the chi‐square (χ^2^) test. Multivariate logistic regression model was used to determine the association of sociodemographic variables and knowledge levels. Descriptive statistics, including frequencies, percentages, means, and standard deviations, were used to summarize the participants’ sociodemographic characteristics and knowledge‐related responses, as appropriate. Knowledge scores were calculated based on the prespecified answer key, with correct responses assigned “1” point and incorrect responses assigned “0” points. The total knowledge score was calculated by summing the scores across the 19 knowledge items, yielding a possible range of 0–19. Participants' knowledge levels were categorized according to the predefined classification criteria. In these models, bivariate analyses were performed to examine associations between participants' sociodemographic characteristics and knowledge levels. Categorical variables were compared using the chi‐square test or Fisher's exact test, as appropriate. Binary logistic regression was used to assess factors associated with good knowledge. Variables considered relevant based on the study objectives and bivariate analyses were evaluated for inclusion in the multivariable model. Adjusted odds ratios (AORs) with 95% confidence intervals (CIs) were calculated to quantify the associations between explanatory variables and good knowledge. A *p* < 0.05 (two‐sided) was considered statistically significant in bivariate analyses. Since the outcome variable “knowledge” is dichotomous, binary logistic regression was used for multivariate analysis for each separately. This method estimates the possibility of the outcome variables while adjusting for multiple factors and produces odds ratios (ORs) with 95% confidence intervals (CIs) as interpretable effect sizes. The number of predictors and model diagnostics were also assessed using Hosmer–Lemeshow goodness‐of‐fit test (*χ* ^2^ = 4.68, df = 8, *p* = 0.790), Omnibus test of model coefficient (*χ* ^2^ = 117.14, df = 3, *p* < 0.001), Nagelkerke's *R* ^2^ value (Nagelkerke *R* ^2^: 41.7%). Before model construction, multicollinearity among predictors was evaluated using the Variance Inflation Factor (VIF). All the variables from this study employed in the model had VIF values less than 3, ranging from < 3 indicating no significant multicollinearity [34]. Therefore, multicollinearity was not a concern in this model.

## 3 Results

Table 2 shows the socio‐demographic characteristic of the respondents. A total of 415 rural residents were enrolled in this study (mean age, 37.56 ± 14.63 years). Participants aged 25–34 years were 29.2%, followed by those under 25 years 20.7% and 15.9% were older than 55 years. Male respondents were 66.5%, and 71.3% of participants were married. With respect to education, 16.4% had received no formal education, 26.0% had completed secondary education, and 27.2% held a graduate or higher degree, respectively. Students constituted the largest occupational category 21.2%, housewives 19.3%, day laborers 14.9%, and farmers 14.0%. A monthly family income below BDT 10,000 was reported by 48.7% of participants. Mean body mass index (BMI) was 22.69 ± 2.99 kg/m^2^, and 51.1% of participants had a normal BMI. Most participants had never smoked (72.0%), and no history of chronic disease was reported among 72.3% of respondents. Consumption of fresh date palm sap was reported by 72.8% of respondents.

**Table 2:** Sociodemographic characteristics of the participants.

Characteristic	Frequency (*N*)	Percentage (%)	Mean ± SD
Age group (years)			37.56 ± 14.63
< 25	86	20.7	
25–34	121	29.2	
35–44	70	16.9	
45–54	72	17.3	
> 55	66	15.9	
Gender			
Male	276	66.5	
Female	139	33.5	
Education level			
No formal education	68	16.4	
Primary	126	30.4	
Secondary	108	26.0	
Graduation and above	113	27.2	
Marital status			
Single	119	28.7	
Married	296	71.3	
Occupation			
Day laborer	62	14.9	
Farmer	58	14.0	
Housewife	80	19.3	
Businessmen	42	10.1	
Student	88	21.2	
Govt. job	37	8.9	
Unemployed	48	11.6	
Monthly family income (BDT)			12782.36 ± 2418.49
< 10,000	202	48.7	
10,000–20,000	165	39.8	
> 20,000	48	11.6	
BMI category			22.69 ± 2.99
Normal weight	212	51.1	
Underweight	21	5.1	
Overweight	162	39.0	
Obesity	20	4.8	
Smoking status			
Never smoke	299	72.0	
Current smoker	116	28.0	
Chronic disease			
Absent	300	72.3	
Present	115	27.7	
Fresh date juice consumption			
No	113	27.2	
Yes	302	72.8	

*Note:* Descriptive analysis was performed. Monthly income was calculated as per the exchange rate on Sunday, March 1, 2026 (1USD = 122.00 BDT).

Abbreviations: BDT, Bangladeshi currency (Taka); BMI, body mass index.

Responses to individual knowledge items are summarized in Table 3. Prior awareness of NiV was reported by 71.1% of participants, and 70.8% were aware that zoonotic transmission from bats to humans is possible. Bats were correctly identified as the natural reservoir of NiV by 54.7% of participants. Bat saliva and excreta were correctly identified as potential sources of infection by 63.6% of respondents. Knowledge of human‐to‐human transmission was also limited. Close contact with an infected individual was correctly identified as a transmission route by 25.8% of participants, and droplet transmission by 36.1%. Awareness was similarly low regarding seasonal occurrence (44.3%), prior outbreaks in Bangladesh (38.1%), district‐level susceptibility (23.9%), and the possibility of asymptomatic infection (20.0%). On average, participants correctly answered approximately eight questions out of 19 questions; the lowest and highest number of questions answered correctly by any individual were 0 and 16, respectively. The *Q*_1_ and *Q*_3_ values of knowledge scores indicate that about one‐fourth and three‐fourth of respondents scored a minimum of 4 and 11, respectively.

**Table 3:** Knowledge statements regarding Nipah virus among the study participants.

Statements	No, *n* (%)	Yes, *n* (%)	Test statistic	*p*‐values
1. Have you ever heard about the Nipah virus?	120 (28.9%)	295 (71.1%)	73.79	0.000
2. Do you know the Nipah virus is life‐threatening?	238 (57.3%)	177 (42.7%)	8.96	0.003
3. Do you know how the Nipah virus is transmitted?	279 (67.2%)	136 (32.8%)	49.27	0.000
4. Do you know which animal is the natural host of the Nipah Virus?	188 (45.3%)	227 (54.7%)	3.66	0.000
5. Do you know there is no proper treatment for the Nipah Virus?	344 (82.9%)	71 (17.1%)	179.58	0.000
6. Have you ever heard of a disease that can be transmitted from bat to people?	121 (29.2%)	294 (70.8%)	72.11	0.000
7. Can close contact with a Nipah‐infected person spread the virus?	308 (74.2%)	107 (25.8%)	97.35	0.000
8. Do you know drinking date palm sap is a common risk factor for infection?	188 (45.3%)	227 (54.7%)	3.66	0.056
9. Do you know the Nipah virus cannot be cured with antibiotics?	338 (81.4%)	77 (18.6%)	164.14	0.000
10. Do you know in which season the prevalence of the Nipah virus is high?	231 (55.7%)	184 (44.3%)	5.32	0.021
11. Do you know about any symptoms of Nipah virus infection?	314 (75.7%)	101 (24.3%)	109.32	0.000
12. Do you know that patients suffering from Nipah virus infection must be isolated?	271 (65.3%)	144 (34.7%)	38.86	0.000
13. Do you know excreta of bats and saliva can infect people?	151 (36.4%)	264 (63.6%)	30.76	0.000
14. The Nipah virus can be transmitted from human to human via droplet infection?	265 (63.9%)	150 (36.1%)	31.86	0.000
15. Nipah virus infection can be asymptomatic.	332 (80.0%)	83 (20.0%)	149.40	0.000
16. The Nipah virus can be passed on during sexual intercourse	231 (55.7%)	184 (44.3%)	5.32	0.000
17. Do you know Bangladesh has seen a severe outbreak of the Nipah Virus?	257 (61.9%)	158 (38.1%)	23.61	0.000
18. Do you know which regions of Bangladesh are more prone to Nipah virus outbreak in our country?	316 (76.1%)	99 (23.9%)	113.46	0.000
19. Do you think date juice must be covered by any net or something to protect from infected animals?	75 (18.1%)	340 (81.9%)	169.21	0.000
Summary statistics of Knowledge score				
Mean ± SD		7.99 ± 3.86		
IQR (Q_1_–Q_3_)		4‐11		
Min.–Max.		0‐16		

*Note:* Descriptive analysis was performed. *p*‐values were obtained using the Chi‐square goodness‐of‐fit test.

Abbreviations: IQR, interquartile range; SD, standard deviation.

Distribution of knowledge according to participant sociodemographic characteristics is presented in Table 4. About 16.4% of participants were reported to have good knowledge, while the remaining 83.6% were classified as having poor knowledge. Knowledge level was significantly associated with age group (*χ* ^2^ = 54.077, *p* < 0.001), gender (*χ* ^2^ = 11.798, *p* < 0.001), education (*χ* ^2^ = 78.057, *p* < 0.001), marital status (*χ* ^2^ = 7.763, *p* = 0.005), occupation (*χ* ^2^ = 48.386, *p* < 0.001), smoking status (*χ* ^2^ = 7.085, *p* = 0.008), presence of chronic disease (*χ* ^2^ = 5.401, *p* = 0.020), and fresh date palm sap consumption (*χ* ^2^ = 21.282, *p* < 0.001). Good knowledge was mostly associated with participants' age between 25 and 34 years (36.4%), female gender (25.2%), graduate or higher education level (42.5%), and government job (45.9%).

**Table 4:** Distribution of knowledge level by sociodemographic characteristics.

	Knowledge			
Characteristic	Poor knowledge (< 65%)	Good knowledge (≥ 65%)	Test statistic	*p*‐value
*N* (%)	**347 (83.6)**	**68 (16.4)**		
Age group (years)			54.077	**< 0.001**
< 25	74 (86.0)	12 (14.0)		
25–34	77 (63.6)	44 (36.4)		
35‐44	64 (91.4)	6 (8.6)		
45–54	67 (93.1)	5 (6.9)		
> 55	65 (98.5)	1 (1.5)		
Gender			11.798	**< 0.001**
Male	243 (88.0)	33 (12.0)		
Female	104 (74.8)	35 (25.2)		
Education			78.057	**< 0.001**
No formal education	65 (95.6)	3 (4.4)		
Primary	119 (94.4)	7 (5.6)		
Secondary	98 (90.7)	10 (9.3)		
Graduation and above	65 (57.5)	48 (42.5)		
Marital status			7.763	**0.005**
Single	90 (75.6)	29 (24.4)		
Married	257 (86.8)	39 (13.2)		
Occupation			48.386	**< 0.001**
Laborer	57 (91.9)	5 (8.1)		
Farmer	57 (98.3)	1 (1.7)		
Housewife	74 (92.5)	6 (7.5)		
Businessmen	34 (81.0)	8 (19.0)		
Student	64 (72.7)	24 (27.3)		
Govt. job	20 (54.1)	17 (45.9)		
Unemployed	41 (85.4)	7 (14.6)		
Monthly family income (BDT)			0.003	0.998
< 10,000	169 (83.7)	33 (16.3)		
10,000–20,000	138 (83.6)	27 (16.4)		
> 20,000	40 (83.3)	8 (16.7)		
BMI category			2.957	0.349
Normal weight	181 (85.4)	31 (14.6)		
Underweight	15 (71.4)	6 (28.6)		
Overweight	135 (83.3)	27 (16.7)		
Obesity	16 (80.0)	4 (20.0)		
Smoking status			7.085	**0.008**
Never smoke	241 (80.6)	58 (19.4)		
Current smoker	106 (91.4)	10 (8.6)		
Chronic disease			5.401	**0.020**
Absent	243 (81.0)	57 (19.0)		
Present	104 (90.4)	11 (9.6)		
Fresh date juice consumption			21.282	**< 0.001**
No	79 (69.9)	34 (30.1)		
Yes	268 (88.7)	34 (11.3)		

*Note:* Chi‐square test was performed; *p* value < 0.05 was considered as statistically significant. Bold values indicate statistical significance (*p* < 0.05).

Factors associated with good knowledge among respondents regarding NiV are presented in Table 5. After adjustment for potential confounders, participants aged 25–34 years had significantly higher odds of good knowledge than those under 25 years (AOR = 2.85, 95% CI: 1.13–7.63; *p* = 0.031). Female participants likewise had higher odds of good knowledge than males (AOR = 2.73, 95% CI: 1.09–7.09; *p* = 0.035). Education was the strongest independent predictor of knowledge: participants with graduate or higher education had more than 10‐fold higher odds of good knowledge than those with no formal education (AOR = 10.70, 95% CI: 2.10–67.70; *p* = 0.007). Occupation was associated with knowledge level; housewives had significantly lower odds of good knowledge than laborers (AOR = 0.14, 95% CI: 0.03–0.76; *p* = 0.022). Fresh date palm sap consumption was significantly associated with knowledge, with consumers showing lower odds of good knowledge than non‐consumers (AOR = 0.34, 95% CI: 0.17–0.68; *p* = 0.003).

**Table 5:** Multivariate logistic regression of factors associated with good knowledge level among the study participants.

Characteristic	AOR	95% CI	*p*‐value
Age group (years)			
< 25	**Reference**	—	
25–34	2.85	1.13, 7.63	**0.031**
35–44	1.54	0.36, 6.45	0.555
45–54	0.94	0.20, 4.26	0.935
> 55	0.20	0.01, 1.67	0.193
Gender			
Male	**Reference**	—	
Female	2.73	1.09, 7.09	**0.035**
Education			
No formal education	**Reference**	—	
Primary	1.83	0.40, 10.4	0.458
Secondary	2.03	0.46, 11.2	0.376
Graduation and above	10.7	2.10, 67.7	**0.007**
Marital status			
Single	**Reference**	—	
Married	0.91	0.33, 2.52	0.850
Occupation			
Laborer	**Reference**	—	
Farmer	0.28	0.01, 2.05	0.276
Housewife	0.14	0.03, 0.76	**0.022**
Businessmen	3.99	0.96, 18.2	0.061
Student	0.33	0.06, 1.88	0.206
Govt. Job	0.86	0.16, 4.76	0.861
Unemployed	1.19	0.24, 5.83	0.829
Monthly family income (BDT)			
< 10,000	**Reference**	—	
10,000–20,000	0.51	0.18, 1.42	0.201
> 20,000	0.25	0.06, 0.99	0.057
BMI category			
Normal weight	**Reference**	—	
Underweight	1.30	0.35, 4.51	0.682
Overweight	0.99	0.47, 2.08	0.970
Obesity	1.26	0.26, 5.17	0.760
Smoking status			
Never smoke	**Reference**	—	
Current smoker	0.63	0.24, 1.56	0.333
Chronic disease			
Absent	**Reference**	—	
Present	1.55	0.59, 4.01	0.368
Fresh date juice consumption			
No	**Reference**	—	
Yes	0.34	0.17, 0.68	**0.003**

*Note:* Omnibus Test of Model Coefficients (*χ* ^2^ = 117.14, df = 23, *p* < 0.001); Cox and Snell R‐squared: 24.6%, Nagelkerke R‐squared: 41.7%; Hosmer–Lemeshow goodness‐of‐fit test (*χ* ^2^ = 4.68, df = 8, *p* = 0.790).

Abbreviations: AOR, adjusted odds ratio; CI, confidence interval.

## 4 Discussion

The present study assessed knowledge level regarding NiV among the rural residents of Jashore District, Bangladesh, and identified factors associated with adequate knowledge. Overall, only 16.4% of participants demonstrated good knowledge, indicating that awareness remains substantially inadequate in this rural population in Bangladesh. This proportion is considerably lower than the 29.2% of respondents with good knowledge reported in a recent nationwide survey conducted across 16 districts of Bangladesh [23], and substantially lower than the 62% and 46% reported by Mokbul et al. (2025) [21] and Islam et al. (2024) [22]. This variation may be due to the extensive focus on rural areas employed in this study. Generally, in rural areas of Bangladesh, educational facilities, access to proper healthcare, and public health awareness are poorer than those of urban areas. Additionally, different questionnaire patterns, knowledge levels, and study samples are also important factors for inconsistency among the studies. A study conducted in India [35] reported that 21% of respondents were at a poor knowledge level regarding NiV, and no respondent was found to be at a good knowledge level.

This study found that the overall knowledge among the respondents was poor, but interestingly, it was also found that more than two‐third (71.1%) of the respondents had heard about NiV. This finding corroborates with the finding of another Bangladeshi study [24], where 69.38% respondents were found with awareness about NiV, indicating that the public awareness increased significantly over time. In contrast with the previous community‐related studies in Bangladesh by Nahar et al. (2015) [8] found only 5% of respondents having heard of NiV. Several factors such as frequent outbreak, mass media, government health awareness intervention played important role for the improvement of respondent's knowledge. However, this study found that the awareness of NiV does not improve sufficient understanding related to the mode of transmission of Nipah virus, clinical features, and preventive actions. This finding supports the findings from Hossain et al. (2025) who reported that though the respondents were aware of NiV but their awareness regarding disease severity and preventive measures were not adequate about NiV infection [23]. Although the majority of infected individuals die from NiV [15], outbreaks frequently garner attention from the media [8]. In addition to using interpersonal and auditory communication in the villages affected by the outbreak, the Bangladeshi government has tried to inform the public about the dangers of consuming raw date palm sap through newspaper announcements and mass media [20]. The danger of NiV transmission would be decreased by consuming less raw date palm sap [36]. Alarmingly, 72.8% of total respondents were included in this consumer raw date palm sap. The possibility of long‐term change may be increased by focusing on increasing awareness of the illness and the risks connected with it. About 67.2% of total respondents do not know how NiV is transmitted, and 74.2% of respondents incorrectly replied to when asked “Can close contact with a Nipah‐infected person spread the virus?”. A study in Bangladesh [37] clearly demonstrated that transmission from person to person was the cause of several Nipah outbreaks. The most significant risk factor for contracting NiV during the outbreak was directly person‐to‐person physical contact with an infected patient, and 51% became ill after having close contact with another Nipah sufferer [38]. This study demonstrated that 70.8% of total respondents were aware about NiV transmission occurs directly from bat to people. Date palm sap infected with bat feces is unsafe for human consumption. People can get infected with NiV and spread it to other family members or contract person. Study conducted regarding NiV transmission and reported bat‐to‐people contamination of virus [9, 39].

In the current study, in real‐life practice, more than two‐third respondents (72.8%) were found to consume fresh date palm juice even though approximately 71% of respondents heard of NiV, 43% knew it as life‐threatening, 71% know bat can transmit diseases, 64% knew bats' excreta and saliva are infectious, and 64% agreed juice must be covered with net. The present study findings are in accordance with Nahar et al. (2015) [8], who found that the consumption of raw date juice was prevalent among the respondents although they knew about its possible health outcomes. Prior qualitative researches observed that consumption of raw date palm juice is rooted within Bengali culture, which is difficult to change, although it is a frequent public health challenge [20, 40]. This is also corroborating with the fact that community engagement behaviors related to high levels of transmission rate remain difficult to provide through traditional health interventions [20, 41]. Khan et al. (2025) [42] observed that many date palm juice collectors were aware of NiV, but they could not adopt preventive actions such as the incorporation of bamboo skirts over the juice collecting pots. The reasons behind this inconsistency were economic barriers, insufficient labor, and decreased yield. In addition, it is a matter of critical public health concern that the current study found more than two‐third of respondents (72.8%) consumed fresh date palm juice, which was higher than the previous nationwide survey research by Hossain et al. (2025) [23]. This is alarming because it was observed that Nipah cases were 25 times more likely than controls to have consumed raw date palm sap [21]. Southwestern Bangladesh, including Jashore, Jhenaidah, Magura, and Kushtia, is vastly enriched with wild date palm trees; hence, raw sap consumption is also highly common there. Therefore, epidemiologically, these areas attract researchers intensely due to recurrent bat‐borne NiV outbreaks [8].

The present study also demonstrated a considerable lack of respondents' knowledge regarding the clinical features of NiV infection. Less than half of the respondents identified the disease as life‐threatening, but the knowledge regarding the signs, lack of proper treatment, inefficacy of antibiotics, isolation of patients, and person‐to‐person transmission was poor. These findings are alarming because the surveillance data reveal that approximately 29% of NiV infections in Bangladesh resulted from person‐to‐person transmission [11]. Additionally, recent reviews suggest to emphasize that no effective vaccine or antiviral therapy is available, so early preparedness and infection prevention comprise the primary strategy for outbreak control [4]. The similar findings in knowledge regarding disease symptoms and transmission have also been found among rural respondents in India and Bangladesh [23, 43], which suggest that insufficient knowledge of the disease is a significant public health concern across South Asia. Educational level was found to be the most important independent factor of knowledge in the present study. Respondents with graduation‐level education or higher were 10 times more likely to possess good knowledge. This result is in accordance with previous studies in Bangladesh which found improved knowledge regarding NiV and other infectious diseases among respondents with higher educational levels [24, 44]. Higher education promotes better access to authentic health information, enhanced health education, and improved understanding of disease prevention methods.

In this study, age was found to be a significantly associated factor with knowledge. It was observed that respondents with 25–34 years had higher knowledge than younger adults. This study findings is similar with the others study finding in Bangladesh regarding NiV [8, 24, 45] that found better knowledge among respondents with 31–40 years compared with other groups of respondents. The reason behind the findings maybe the social activeness, economic independence, and exposure to various information sources, which are responsible for greater awareness of diseases among this age group. However, it also raises questions about possible carelessness on the part of younger generations with regard to the dangers of ingesting raw date sap.

Notably, female respondents showed remarkably higher levels of good knowledge than male respondents after adjusting for possible confounding variables. This study finding is aligned with the other study findings in Bangladesh [25, 46] and India [31]. This study finding is inconsistent with the study by Mokbul et al. (2025) [21], who found poor knowledge among the female participants. The dissimilarities may be due to different study settings, opportunities for education, and availability of proper health guidelines. Respondents' marital status and occupation were significantly associated (*p* < 0.05) with good knowledge level. Respondents with stable income source showed good level of knowledge than those are not have stable income source. The possible reason behind the findings economic independence may influence the greater awareness of NiV infection among the respondents. This finding is in line with pervious study in Bangladesh [23, 24]. Respondents not having a smoking habit and no reported chronic disease were significantly associated (*p* < 0.05) with good knowledge regarding NiV. Possible explanation behind this nonsmoker's individual and respondents without chronic disease may be more health‐conscious and have better access to health information.

These findings have important public health implications. Bangladesh experiences substantial outbreaks of NiV, and the absence of an effective licensed vaccine or specific antiviral treatment makes prevention a fundamental component of disease control [4, 47]. From a broader One Health perspective, effective NiV preparedness requires integration of community‐level prevention and risk communication with surveillance, cross‐sectoral coordination, diagnostic capacity, and health‐system preparedness [48]. The low level of community knowledge identified in the present study therefore represents an important community‐level component of the broader preparedness gap. Intervention strategies should specifically emphasize the risks associated with consumption of raw date palm sap, recognition of early symptoms, appropriate preventive measures to reduce transmission, and the importance of timely medical care. Earlier intervention‐based researchers have found that community‐based education program specially focusing on the localities can greatly improve knowledge about NiV. Implementation of health education program for school‐going children increased awareness regarding raw date juice consumption and its associated disease transmission, personal hygiene practices, and disease prevention practices [49, 50]. This study's findings recommended that future intervention program should combine health education with culturally acceptable and affordable preventive practices to maximize sustainable effectiveness.

## 5 Conclusion

The study found that the knowledge level among the rural residents of Jashore district was not adequate with only 16.4% having good knowledge. Though most respondents heard about NiV and were aware of bats as the main source of infection, the knowledge gaps persisted regarding disease transmission, clinical manifestations, and treatment. The study also found that age, gender, educational level, occupation, and fresh date sap consumption were significantly associated with knowledge levels of the respondents. Respondents aged 25–34 years, females, and respondents with graduate‐level education or above were found with good knowledge. Housewives and respondents consuming fresh date palm sap had a low level of adequate knowledge. The study findings reveal the urgency for targeted, community‐level health education and awareness interventions in rural areas of Bangladesh, focusing among respondents with a low level of education and those who consume raw date palm sap. Public health interventions should include the mode of transmission, diagnosis, and preventive measures of NiV. Extensive community awareness through community healthcare providers, stakeholders, and mass media may be effective for better preparedness and reducing the risk of NiV outbreaks in Bangladesh. Moreover, longitudinal studies with knowledge, attitude, and practice levels are recommended to provide a better understanding of the overall evidence‐based prevention studies.

## 6 Strength and Limitations

The study has several strengths. First, one of the key strengths of this study lies in its originality. This is the first community‐based study with multistage sampling in Jashore, Bangladesh. Second, the study's sample size (*n* = 415) exceeded the minimum required, enhancing the precision of the statistical analysis. Third, the questionnaire was developed from an extensive literature review, and a pilot test was conducted before the final survey. The acceptable internal consistency also supports the reliability of the knowledge scale of the questionnaire. Finally, the AOR from the multivariable logistic regression enhanced the identification of confounding factors of good knowledge. Moreover, a comprehensive statistical analysis was done to describe the outcome variable.

Although it has several strengths, the study still has some limitations. First, a cross‐sectional study does not determine the effective associations between respondents' sociodemographic information and knowledge level of the NiV. Moreover, as Jashore was purposively selected for this study, the sample size may not fully represent the rural respondents of the study area. Second, this study only highlights the rural respondents in Jashore; this is not generalized to urban populations or other regions of Bangladesh. Third, the knowledge questions may be affected by recall bias or social desirability bias, as they were assessed by self‐reported responses. Finally, the study only evaluated the knowledge level among the respondents without assessing the attitude and practice levels among the respondents. This limits the comprehensive understanding of community preparedness and prevention measures against the NiV.

## Author Contributions

**Tamsel Ahammed:** conceptualization, investigation, writing – original draft, writing – review and editing, visualization, validation, methodology, software, project administration, data curation, resources, formal analysis. **Md. Ahsan Habib:** conceptualization, investigation, writing – original draft, writing – review and editing, visualization, validation, methodology, software, formal analysis, project administration, data curation, resources. **Md. Moshiuzzaman Adnan:** formal analysis, data curation, validation, visualization, methodology. **Sabrina Zaman:** conceptualization, investigation, writing – original draft, writing – review and editing, visualization, methodology, validation, software, formal analysis, data curation, resources. **Natasha Kundu:** writing – original draft, writing – review and editing. **Mst Sumaya Binta Bilkis:** data curation. **Kazi Nazifa Anzum Setu:** data curation. **Md. Lokman Hossain:** data curation. **Md. Rakibul Hassan Tarek:** data curation. **Susmita Roy Chowdhury:** writing – original draft, writing – review and editing, methodology, formal analysis, validation, visualization, resources, investigation, software. **Md. Shamimuzzaman:** writing – review and editing, formal analysis. **Rajib Kanti Roy:** conceptualization, investigation, writing – original draft, funding acquisition, visualization, validation, methodology, writing – review and editing, software, formal analysis, project administration, data curation, supervision, resources.

## Funding

The authors have nothing to report.

## Ethical Statement

Ethical approval for this study was obtained from the Ethical Review Committee, Faculty of Biological Science and Technology, Jashore University of Science and Technology, Jashore, Bangladesh (Approval No. ERC/FBST/JUST/2026‐305). Written informed consent was obtained from all participants before data collection. Participants were informed about the objectives of the study, the voluntary nature of participation, their right to withdraw from the study at any time without consequence, and the confidentiality of their responses. No personally identifiable information was collected, and all data were used solely for research purposes in accordance with the principles of the Declaration of Helsinki. Written consent was obtained from every respondent.

## Consent

The participants have provided written informed consent for publication of this study.

## Conflicts of Interest

The authors declare no conflicts of interest.

## Declaration of Generative AI and AI‐Assisted Technologies in the Writing Process

The authors declare that no generative AI or AI‐assisted technologies were used in the writing of this manuscript.

## Transparency Statement

The corresponding author Rajib Kanti Roy affirms that this manuscript is an honest, accurate, and transparent account of the study being reported; that no important aspects of the study have been omitted; and that any discrepancies from the study as planned (and, if relevant, registered) have been explained.

The corresponding author Rajib Kanti Roy affirm that this manuscript is an honest, accurate, and transparent account of study being reported.

## Data Availability

All authors have read and approved the final version of the manuscript. Rajib Kanti Roy had full access to all of the data in this study and takes complete responsibility for the integrity of the data and the accuracy of the data analysis. All the data that support the study will be available upon direct request to the corresponding author.
